# Supplementary material for: Paraquat is an agonist of STIM1 and increases intracellular calcium levels
Source: Commun Biol. 2022 Oct 30;5:1151. doi: 10.1038/s42003-022-04130-0 (PMC9618025; doi:10.1038/s42003-022-04130-0)
Supplement: Supplementary file 2 — Supplementary figure [file 42003_2022_4130_MOESM2_ESM.pdf]

SUPPLEMENTARY INFORMATION

Supplementary Fig. 1 (a) FACS analysis of intracellular calcium levels by Fluo-3 staining in A549 cells. The representative image shows cells treated with 5 μM or 10μM SKF-96365 (SKF) as indicated, affiliated to Fig. 2b. **(b, c)** Calcium imaging of A549 cells treated with 0 or 800 μM PQ, addition with 1 μM thapsigargin and followed by the addition of 2 mM $CaCl_2$ (b), or 1 μM thapsigargin addition with 800 μM PQ and followed by the addition of 2 mM $CaCl_2$ (c). TG, thapsigargin. Mean ± sem. **(d)** A549 cells treated with 0 or 800 μM PQ, addition with 5 mM $SrCl_2$. Mean ± sem. **(e)** Chemical formula of the two PQ-Biotin utilized in the study.

Figure S1

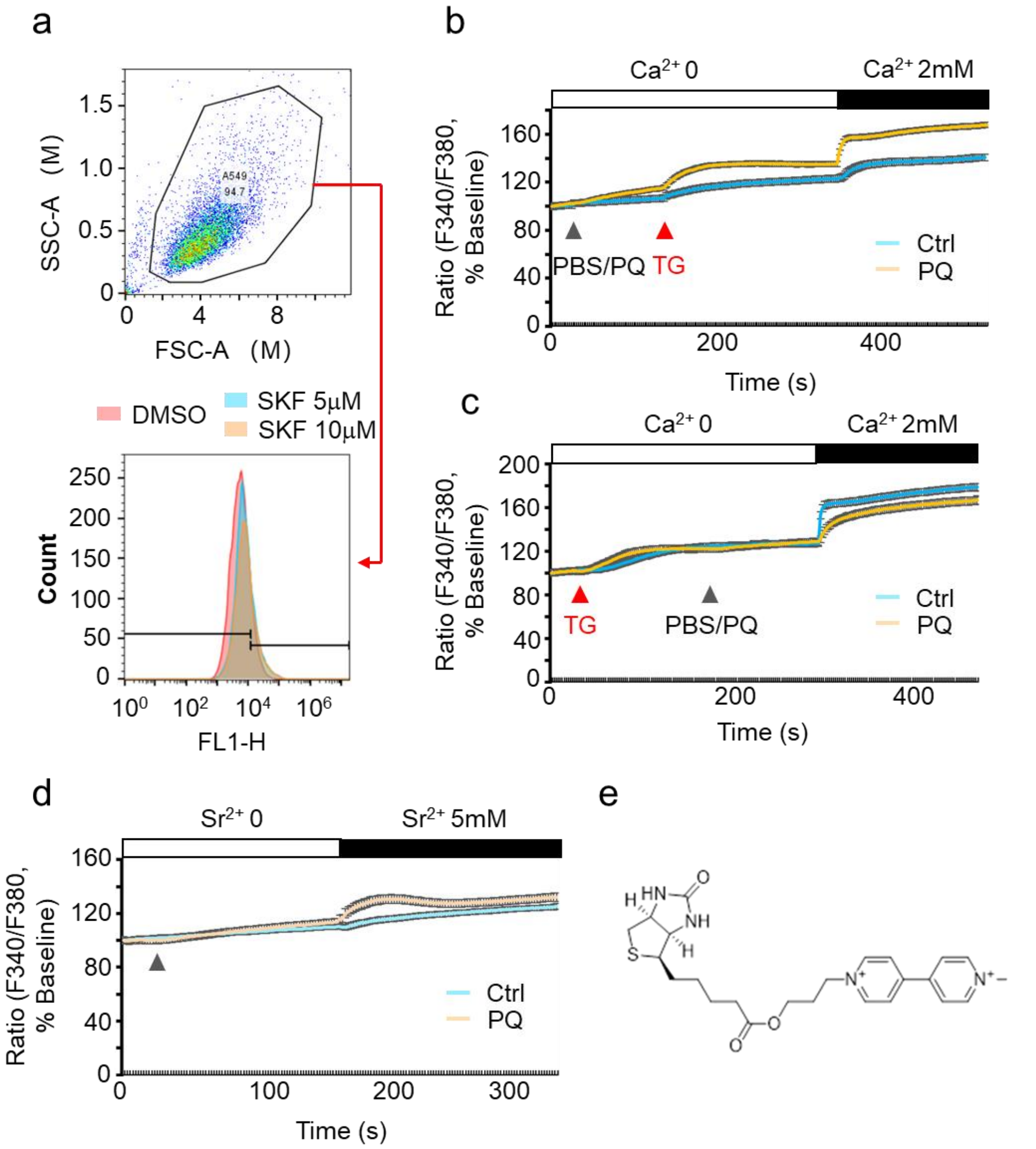
a
SSC-A (M)
1.5
1.0
0.5
0
A549
94.7
0
4
8
FSC-A (M)
DMSO
SKF 5μM
SKF 10μM
Count
250
200
150
100
50
0
10^0
10^2
10^4
10^6
FL1-H
b
Ca2+ 0
Ca2+ 2mM
Ratio (F340/F380, % Baseline)
160
120
80
40
0
PBS/PQ
TG
Ctrl
PQ
0
200
400
Time (s)
c
Ca2+ 0
Ca2+ 2mM
200
160
120
80
40
0
TG
PBS/PQ
Ctrl
PQ
0
200
400
Time (s)
d
Sr2+ 0
Sr2+ 5mM
Ratio (F340/F380, % Baseline)
160
120
80
40
0
Ctrl
PQ
0
100
200
300
Time (s)
e
H
HN
O
NH
S
H
O
O
N+
N+

Supplementary Fig. 2 Original full blots of western blots.

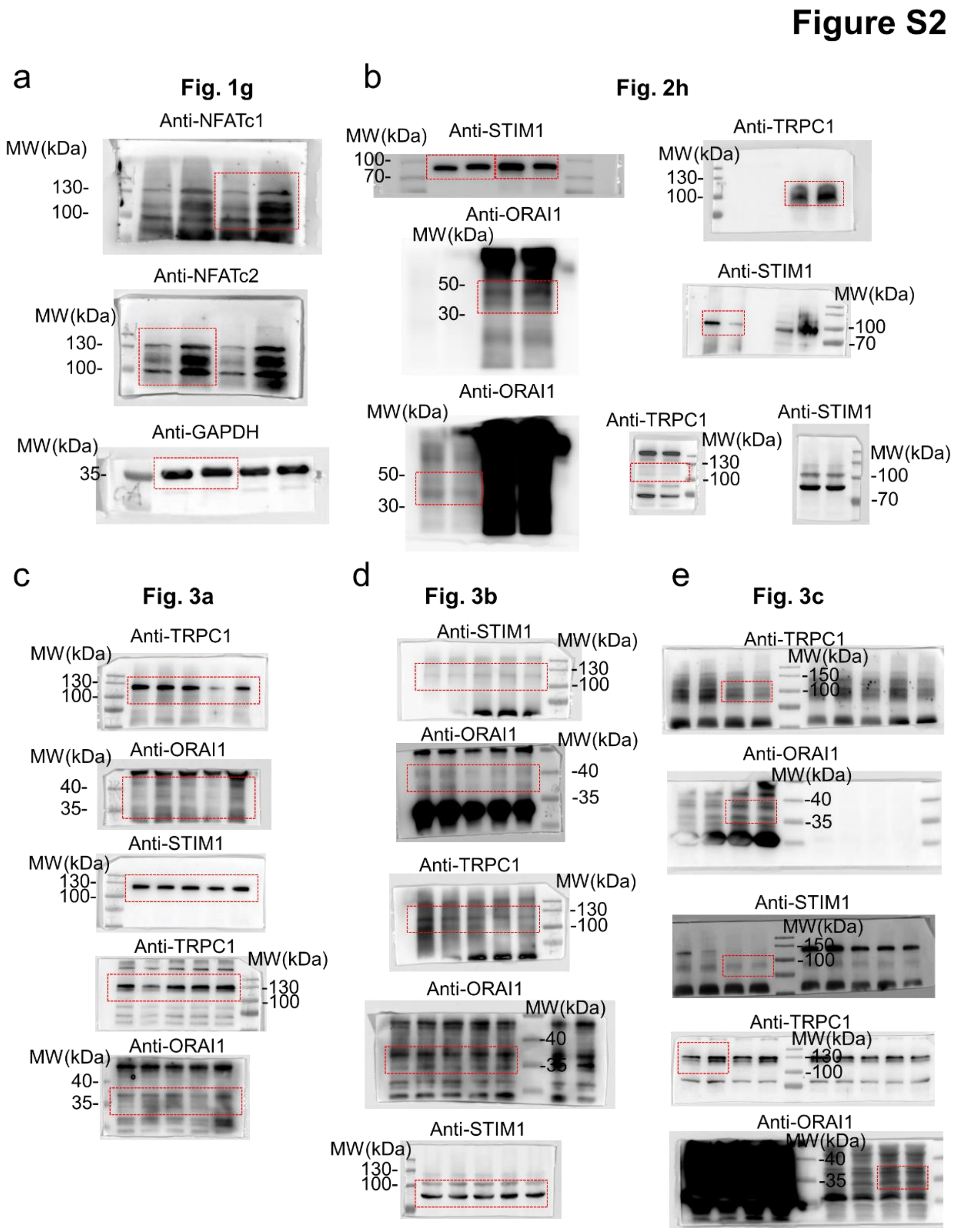

Figure S2

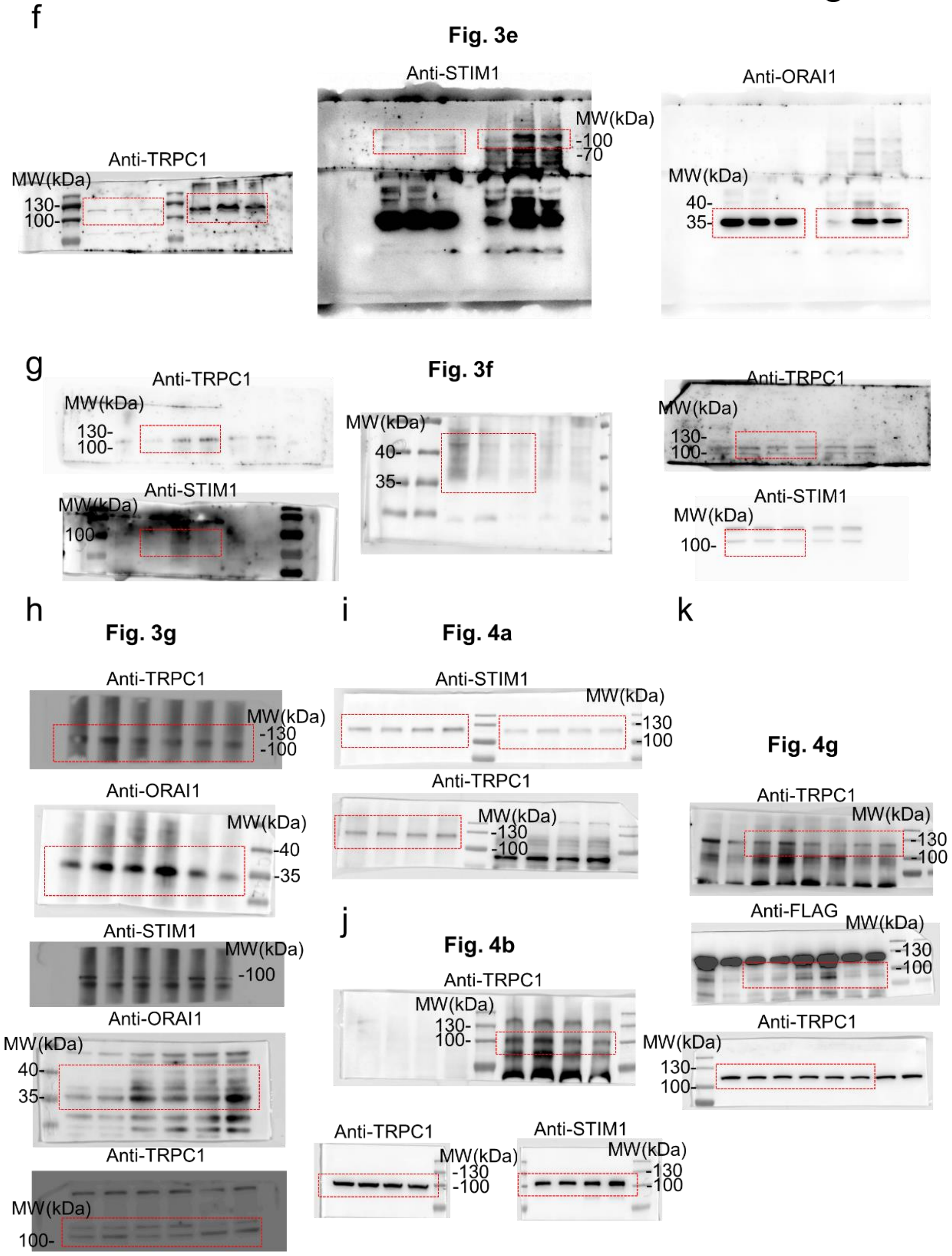
f
Fig. 3e
Anti-TRPC1
MW(kDa)
130-
100-
Anti-STIM1
MW(kDa)
-100
-70
Anti-ORAI1
MW(kDa)
40-
35-
g
Anti-TRPC1
MW(kDa)
130-
100-
Anti-STIM1
MW(kDa)
100
Fig. 3f
MW(kDa)
40-
35-
Anti-TRPC1
MW(kDa)
130-
100-
Anti-STIM1
MW(kDa)
100-
h
Fig. 3g
Anti-TRPC1
MW(kDa)
-130
-100
Anti-ORAI1
MW(kDa)
-40
-35
Anti-STIM1
MW(kDa)
-100
Anti-ORAI1
MW(kDa)
40-
35-
Anti-TRPC1
MW(kDa)
100-
i
Fig. 4a
Anti-STIM1
MW(kDa)
-130
-100
Anti-TRPC1
MW(kDa)
-130
-100
j
Fig. 4b
Anti-TRPC1
MW(kDa)
130-
100-
Anti-TRPC1
MW(kDa)
-130
-100
Anti-STIM1
MW(kDa)
-130
-100
k
Fig. 4g
Anti-TRPC1
MW(kDa)
-130
-100
Anti-FLAG
MW(kDa)
-130
-100
Anti-TRPC1
MW(kDa)
130-
100-

Figure S2

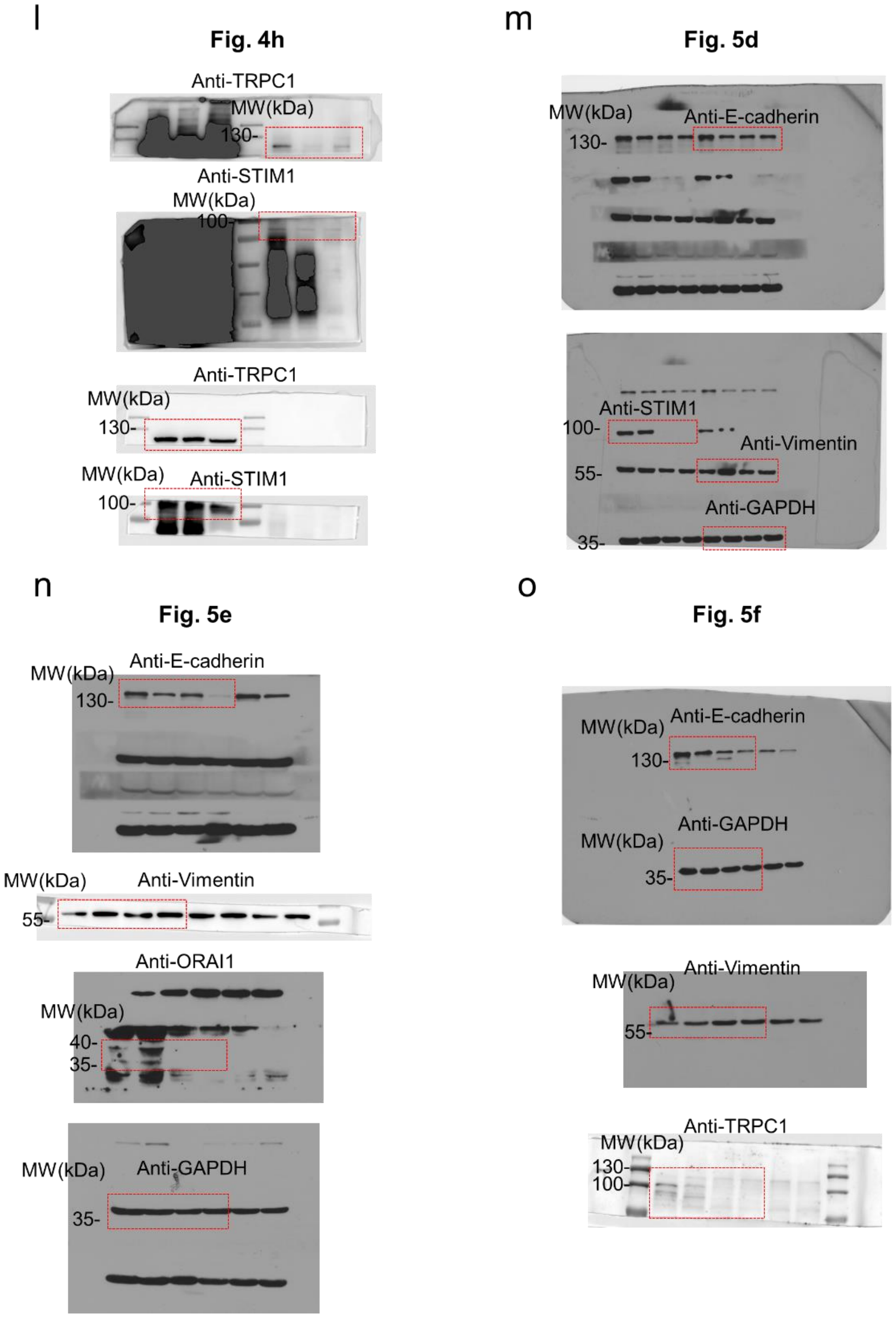

l
Fig. 4h
Anti-TRPC1
MW(kDa)
130-
Anti-STIM1
MW(kDa)
100-
Anti-TRPC1
MW(kDa)
130-
MW(kDa)
Anti-STIM1
100-
m
Fig. 5d
MW(kDa)
Anti-E-cadherin
130-
Anti-STIM1
100-
Anti-Vimentin
55-
Anti-GAPDH
35-
n
Fig. 5e
MW(kDa)
Anti-E-cadherin
130-
MW(kDa)
Anti-Vimentin
55-
Anti-ORAI1
MW(kDa)
40-
35-
MW(kDa)
Anti-GAPDH
35-
o
Fig. 5f
MW(kDa)
Anti-E-cadherin
130-
Anti-GAPDH
MW(kDa)
35-
Anti-Vimentin
MW(kDa)
55-
Anti-TRPC1
MW(kDa)
130-
100-

Supplementary Table 1. Information on PCR Primers.

Table S1. PCR primers

Gene		Sequence
Stim1	sense	5' TGGTGAGGATAAGCTCATCAGC 3'
	antisense	5' CAGCACAGTCCCTGTCATGG 3'
Orai1	sense	5' GGACGCTGACCACGACTAC 3'
	antisense	5' GGGACTCCTTGACCGAGTT 3'
Trpc1	sense	5' TAGTGACGAGCCTCTTGACAA 3'
	antisense	5' CTGGCAGTTAGACTGGGAGA 3'
Trpc3	sense	5' AGAATGACTATCGGAAGCTCTCC 3'
	antisense	5' GGCAAGTTTGACACGACTTAATG 3'
Trpc4	sense	5' AAAAGCCCACTTGGACTGTTC 3'
	antisense	5' GACCTGTCGATGTGCTGAGAG 3'
Trpc5	sense	5' CACACCGGACATCACTCCC 3'
	antiense	5' ACCTCTGAACTAGACACACACT 3'
Trpc6	sense	5' GTTATGTTCGGATTGTGGAAGCA 3'
	antisense	5' ACATCATGGGAGAACCGTGTC 3'
Trpc7	sense	5' CGAGAAACAGCGGAAAGACTC 3'
	antisense	5' TCTGGCTAACTCGTTGCTGAG 3'
Trpv1	sense	5' CAGGCTCTATGATCGCAGGAG 3'
	antisense	5' TTTGAACTCGTTGTCTGTGAGG 3'
Trpv2	sense	5' GACTTCCAGAGTACCTGAGCA 3'
	antisense	5' GCAGGCATTGACTCCGTCC 3'
Trpv3	sense	5' AGGCTTCTACTTCGGTGAGAC 3'
	antisense	5' AGGGCGTGAAGGATGTTGTTG 3'
Trpv4	sense	5' CTACGGCACCTATCGTCACC 3'
	antisense	5' TTAGGCGTTTCTTGTGGGTCA 3'
Trpv5	sense	5' GAGCTGGTGAGCATCGTTG 3'
	antisense	5' CCCCAAGAATCGTCTTTCCAA 3'
Trpv6	sense	5' AGGACCAATAACCGCACGAG 3'
	antisense	5' ATGTCTGGAACCTCTACCAGC 3'
Gapdh	sense	5' ACCCAGAAGACTGTGGATGG 3'
	antisense	5' TTCAGCTCAGGGATGACCTT 3'
E-cadherin	sense	5′ TTCCTCCCAATACATCTCCC 3′
	antisense	5′ TTGATTTTGTAGTCACCCACC 3′
Vimentin	sense	5′ CTCTTCCAAACTTTTCCTCCC 3′
	antisense	5′ AGTTTCGTTGATAACCTGTCC 3′
