# Supplementary material for: Paraquat is an agonist of STIM1 and increases intracellular calcium levels
Source: Commun Biol. 2022 Oct 30;5:1151. doi: 10.1038/s42003-022-04130-0 (PMC9618025; doi:10.1038/s42003-022-04130-0)
Supplement: Supplementary file 3 — Description of Additional Supplementary Data [file 42003_2022_4130_MOESM3_ESM.docx]

**Description of Additional Supplementary Files**

**File name:** Supplementary Data 1

**Description:** The numerical data of the graphs in the paper
