# Supplementary material for: Paraquat is an agonist of STIM1 and increases intracellular calcium levels
Source: Commun Biol. 2022 Oct 30;5:1151. doi: 10.1038/s42003-022-04130-0 (PMC9618025; doi:10.1038/s42003-022-04130-0)
Supplement: Supplementary file 5 — Reporting Summary [file 42003_2022_4130_MOESM5_ESM.pdf]

nature portfolio

Corresponding author(s): Zhengfeng Yang
Last updated by author(s): Oct 9, 2022

Reporting Summary

Nature Portfolio wishes to improve the reproducibility of the work that we publish. This form provides structure for consistency and transparency in reporting. For further information on Nature Portfolio policies, see our Editorial Policies and the Editorial Policy Checklist.

Statistics

For all statistical analyses, confirm that the following items are present in the figure legend, table legend, main text, or Methods section.

n/a	Confirmed	
☐	☒	The exact sample size (*n*) for each experimental group/condition, given as a discrete number and unit of measurement
☐	☒	A statement on whether measurements were taken from distinct samples or whether the same sample was measured repeatedly
☐	☒	The statistical test(s) used AND whether they are one- or two-sided<br>*Only common tests should be described solely by name; describe more complex techniques in the Methods section.*
☒	☐	A description of all covariates tested
☒	☐	A description of any assumptions or corrections, such as tests of normality and adjustment for multiple comparisons
☐	☒	A full description of the statistical parameters including central tendency (e.g. means) or other basic estimates (e.g. regression coefficient) AND variation (e.g. standard deviation) or associated estimates of uncertainty (e.g. confidence intervals)
☒	☐	For null hypothesis testing, the test statistic (e.g. *F*, *t*, *r*) with confidence intervals, effect sizes, degrees of freedom and *P* value noted<br>*Give P values as exact values whenever suitable.*
☒	☐	For Bayesian analysis, information on the choice of priors and Markov chain Monte Carlo settings
☒	☐	For hierarchical and complex designs, identification of the appropriate level for tests and full reporting of outcomes
☒	☐	Estimates of effect sizes (e.g. Cohen's *d*, Pearson's *r*), indicating how they were calculated

Our web collection on statistics for biologists contains articles on many of the points above.

Software and code

Policy information about availability of computer code

Data collection	Images and data were collected by instruments described in Materials and Methods.
Data analysis	Data were analyzed by Microsoft Excel 2019 and GraphPad Prism 7.0.

For manuscripts utilizing custom algorithms or software that are central to the research but not yet described in published literature, software must be made available to editors and reviewers. We strongly encourage code deposition in a community repository (e.g. GitHub). See the Nature Portfolio guidelines for submitting code & software for further information.

Data

Policy information about availability of data

All manuscripts must include a data availability statement. This statement should provide the following information, where applicable:

- Accession codes, unique identifiers, or web links for publicly available datasets
- A description of any restrictions on data availability
- For clinical datasets or third party data, please ensure that the statement adheres to our policy

All data of this study are available from the corresponding authors upon the reasonable request.

Field-specific reporting

Please select the one below that is the best fit for your research. If you are not sure, read the appropriate sections before making your selection.

☒ Life sciences ☐ Behavioural & social sciences ☐ Ecological, evolutionary & environmental sciences

For a reference copy of the document with all sections, see nature.com/documents/nr-reporting-summary-flat.pdf

Life sciences study design

All studies must disclose on these points even when the disclosure is negative.

Sample size	Following with the well-established methodologies, the number of samples in each experiment was equal or more than three, as indicated.
Data exclusions	No data were excluded from the analyses.
Replication	All of the experiments were performed at least with technical triplicates and biological replicates.
Randomization	The samples were randomly allocated.
Blinding	Images were captured by one researcher and analyzed by another researcher.

Reporting for specific materials, systems and methods

We require information from authors about some types of materials, experimental systems and methods used in many studies. Here, indicate whether each material, system or method listed is relevant to your study. If you are not sure if a list item applies to your research, read the appropriate section before selecting a response.

Materials & experimental systems

n/a	Involved in the study	
☐	☒	Antibodies
☒	☐	Eukaryotic cell lines
☒	☐	Palaeontology and archaeology
☒	☐	Animals and other organisms
☒	☐	Human research participants
☒	☐	Clinical data
☒	☐	Dual use research of concern

Methods

n/a	Involved in the study	
☒	☐	ChIP-seq
☐	☒	Flow cytometry
☒	☐	MRI-based neuroimaging

Antibodies

Antibodies used	Anti-FLAG (Abmart, M20008, 324085), Anti-Myc (Affinity, T0052, 17j0232), Anti-STIM1 (Cell Signaling Technology, 5668, 3), Anti-ORAI1 (Santa Cruz, sc-377281, H0219), Anti-TRPC1 (Proteintech, 19482, 00025008), Anti-E-cadherin (Cell Signaling Technology, 3195S, 13), Anti-Vimentin (Cell Signaling Technology, 5741S, 6), Anti-GAPDH (Proteintech, 60004-1-1g, 10013030), Anti-NFATc1 (Santa Cruz, sc7294, C0719), and NFATc2 (Santa Cruz, sc7296, J1317), 488-conjugated goat anti-mouse secondary antibody (Thermo, A32723, VC300588) or 488-conjugated goat anti-rabbit secondary antibody (Bioss, bs-0295G, J03276696).
Validation	All of the antibodies used in the study were followed with the manufacturer's instructions. The application information was included in Materials and Methods.

Flow Cytometry

Plots

Confirm that:

☒ The axis labels state the marker and fluorochrome used (e.g. CD4-FITC).

☒ The axis scales are clearly visible. Include numbers along axes only for bottom left plot of group (a 'group' is an analysis of identical markers).

☒ All plots are contour plots with outliers or pseudocolor plots.

☒ A numerical value for number of cells or percentage (with statistics) is provided.

Methodology

Sample preparation	A549 cells, MLE 12 cells or WI-38 cells were seeded in a six-well plate with a density of 1×106 cells/dish in complete growth medium. The cells were then incubated with 2 uM Fluo-3/AM (Beyotime, S1056) diluted in Hanks' Balanced Salt Solution (Beyotime, C0218) for 30 min at 37°C in the dark. The stained cells were then dissociated with trypsin, resuspended with HBSS and collected to detect the presence of [Ca2+]i using a FACScan flow cytometer (BD Accuri™ C6 Plus, NJ, USA).
Instrument	BD Accuri™ C6 Plus
Software	FlowJo 10
Cell population abundance	The cells were labeled with 2uM Fluo-3 AM and 10,000 cells were recorded in each sample for further analysis.
Gating strategy	FSC/SSC gates the lived cell population and the Fluo-3 signals (FL1-H channel) were recorded. To analyze the relevant intracelluar calcium levels, Ctrl cells with around 10% signals in A549 cells were defined as the boundary for further analysis.

☒ Tick this box to confirm that a figure exemplifying the gating strategy is provided in the Supplementary Information.
